# Low-Cost Battery-Powered and User-Friendly Real-Time Quantitative PCR System for the Detection of Multigene

**DOI:** 10.3390/mi11040435

**Published:** 2020-04-21

**Authors:** Junru An, Yangyang Jiang, Bing Shi, Di Wu, Wenming Wu

**Affiliations:** 1 State Key Laboratory of Applied Optics, Changchun Institute of Optics, Fine Mechanics and Physics (CIOMP), Chinese Academy of Sciences, Changchun 130033, China; anjunru18@mails.ucas.ac.cn (J.A.); jiangyy1994@foxmail.com (Y.J.); shibing16@mails.ucas.ac.cn (B.S.); wudi16@mails.ucas.ac.cn (D.W.); 2 University of Chinese Academy of Sciences (UCAS), Beijing 100049, China

**Keywords:** quantitative real-time PCR, fluorescence detection, microchip

## Abstract

Real-time polymerase chain reaction (PCR) is the standard for nucleic acid detection and plays an important role in many fields. A new chip design is proposed in this study to avoid the use of expensive instruments for hydrophobic treatment of the surface, and a new injection method solves the issue of bubbles formed during the temperature cycle. We built a battery-powered real-time PCR device to follow polymerase chain reaction using fluorescence detection and developed an independently designed electromechanical control system and a fluorescence analysis software to control the temperature cycle, the photoelectric detection coupling, and the automatic analysis of the experimental data. The microchips and the temperature cycling system cost USD 100. All the elements of the device are available through open access, and there are no technical barriers. The simple structure and manipulation allows beginners to build instruments and perform PCR tests after only a short tutorial. The device is used for analysis of the amplification curve and the melting curve of multiple target genes to demonstrate that our instrument has the same accuracy and stability as a commercial instrument.

## 1. Introduction

Polymerase chain reaction (PCR) is a molecular reaction used to amplify a specific segment of DNA [1,2,3]. PCR plays an important role in many fields, including pathogen diagnosis, bioprospecting, and environmental protection [4,5,6,7,8,9,10,11,12]. In traditional PCR, the detection of amplified products largely relies on electrophoretic gel analysis [3], which is time-consuming and makes it difficult to quantify the target molecules [13]. Therefore, fluorescent quantitative PCR (qPCR) is widely used to conveniently quantify the amplified products [14,15,16,17,18]. Even now, qPCR is still the gold standard for nucleic acid detection. 

In recent years, many commercial PCR instruments, including the Applied Biosystems (ABI) PRISM^®^ 7900HT and Bio-Rad iQ5 systems, have been successfully brought to the market. These instruments have a good detection performance, but real-time PCR is almost 10 times more expensive than ordinary PCR platforms due to its high sensitivity and signal reporting method [19,20,21,22,23]. The cost of these commercial quantitative PCR devices is relatively high, which is an issue for resource-limited areas. Furthermore, most devices need an external power supply which greatly limits their use. For these reasons, many researchers are developing low-cost real-time fluorescent quantitative PCR systems [23,24]. However, there are several challenges, such as making PCR chips that do not create bubbles during the reaction. Polymerase chain reaction requires a denaturation temperature of 95 °C to uncoil the double-stranded DNA, which is very close to the boiling point of water. This causes bubbles to form in the microfluidic chips and the reaction to fail [25,26]. It is the main problem to be solved by microfluidic engineers. The surface is generally treated by chemical vapor deposition (CVD) and physical vapor deposition (PVD) to fabricate a “bubble-free” chip [27,28]. However, the surface of the chip treated by such methods often denatures after a period of time, and bubbles are generated when the chip is used again. Therefore, these chips need to be used as soon as possible after processing, which limits their application [23,29,30].

Another challenge is to develop a control system with no technical barriers and as little hardware as possible to achieve the coupling between the temperature cycle, the optical imaging, and the automatic analysis of the corresponding data. Although many homemade control systems have been proposed, most of them need multiple customized hardware to achieve their goal [31]. Hardware and software customizations not only increase the cost of the PCR equipment, but also establish a technical barrier, which affects the popularization of the technology. In a previous article, we proposed a method to achieve this through free software that is open access on the web [29]. However, we used an integrated circuit to realize the coupling between the temperature cycle and the photoelectric imaging. The use of custom integrated circuits has increased the technical barriers. Moreover, several manual steps were still needed in processing the fluorescent images, the reading, and the analysis of the fluorescent data. 

The system consists of a built-in battery, a thermoelectric cooler (TEC) for temperature cycling, and a camera. After connecting the device to a laptop using two serial ports, the automation of the quantitative PCR can be realized. There are no customized parts and all elements can be purchased from public sources. The control script is coded in Python and can be written with basic programming knowledge. We also wrote a script for the automatic temperature control and the automatic analysis of the results to display the PCR amplification curve to further improve the efficiency of the experiment. A homemade chip to amplify Avian Influenza A (H7N9) and pGEM-3Zf (+)1 by using this device was developed to validate our instrument against commercial qPCR instruments priced at over USD 50,000. This further verified the accuracy of our system.

## 2. Materials and Methods 

### 2.1. Materials and Reagents

The PCR regent was composed of 1× Premix Ex Taq (TaKaRa Biotechnology (Dalian) Co., Ltd, Dalian, China), 0.075 U μL^−1^ TaKaRa Ex Taq, 0.6 mg mL^−1^ Bovine serum albumin V (AS25483, AMEKO, Shanghai, China), 0.5 μM forward and reverse primers (Genewiz, Suzhou, China).

### 2.2. Instrument Build

The system consisted of three parts: a temperature circling system, an optical detection system, and a control analysis system. 

The temperature circulation system included a TEC (tec1-12705), a fan (90 mm × 90 mm, DC24V, Guangdong, China), a light emitting diode (LED, Cree-xpe 470–480 nm, Shenzhen, China), a camera (Canon 7D), a thermostat (TCM1030, Chengdu, China), a heat sink(69 mm × 69 mm × 36 mm, Shenzhen, China), and a temperature sensor (PT1000A, Zhengzhou, China).

In the Python scripts, the number of cycles, the high and low temperatures, and the time as well as the light times could be user-entered values through a graphical user interface (GUI). It also achieved the coupling of the temperature cycle, lighting time, and the optical imaging.

**Figure 1:**
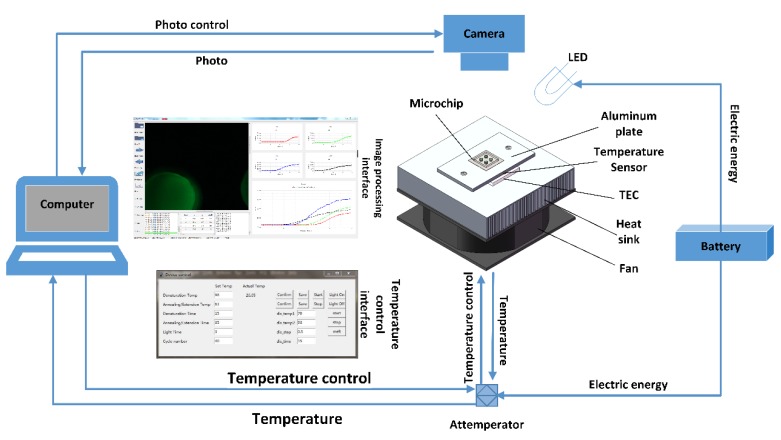
Overview of the fluorescent PCR system. The system is powered by lithium batteries. The temperature cycle of the thermoelectric cooler (TEC) is controlled by a computer. The fluorescence information is collected by a camera. Then, the computer processes the collected information and draws a curve.

In the system, the TEC was used as the heat source and an aluminum plate was used to fix the TEC to a heat sink. A temperature sensor was embedded in the aluminum plate to measure the temperature. We used thermal grease to bind the TEC and the aluminum plate to improve the heat conduction. We fixed a fan under the heat sink to speed up the cooling rate of the system and increase the temperature ramps. The thermostat was used to control temperature. A structural drawing of the device is shown in Figure 1.

### 2.3. Microchip Fabrication

**Figure 2:**
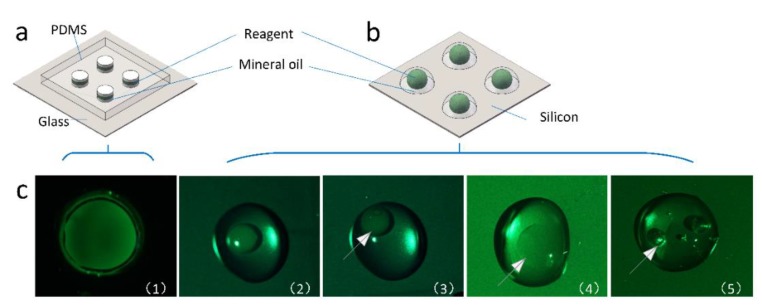
Comparison of the new and previous chip designs. (**a**): current microchips. (**b**): the previous microchips. (**c**): issues with the previous microfluidic chip: (1) droplet after the reaction on the homemade chip, (2) droplet before the reaction, (3) off-center droplet during the injection, (4) sample evaporation during the reaction, and (5) bubbles forming during the reaction.

Polydimethylsiloxane (PDMS, DC184, Dow Corning, Elizabethtown, KY, USA) and a glass piece (20 mm × 20 mm × 0.13 mm) were used for the fabrication. First, the silicone elastomer and the curing agent were mixed in a 10:1 proportion (22 g in total). The mixture was then poured into a Petri dish (100 mm × 100mm). Because of the appearance of bubbles during mixing, the mixture had to be placed in a vacuum chamber (−0.8 $\mathrm{kg}/{\mathrm{cm}}^{2}$) for 30 min. After taking it out of the vacuum chamber, the mixture was left standing until it solidified. The process can be accelerated if the petri dish is placed in an oven at 60 °C. The PDMS made was 1.40 mm thick. After solidification, the PDMS was cut into small blocks (18 mm × 18 mm) with a knife. Then, four transparent holes were cut in the blocks with a puncher (d = 2.5 mm). Next, the glass piece and the PDMS block were placed in plasma cleaner for 40 s to be bonded [32]. Figure 2a shows a structural drawing of the microchip.

### 2.4. PCR Amplification

To verify the applicability of our PCR device, we analyzed pGEM-3Zf (+)1 samples with different concentrations. We made 10-fold serial dilutions of the stock DNA over four orders of magnitude. The sample (20 μL) contained: 3 μL of TaKaRa Ex Taq, 10  μL Premix Taq, 2  μL H7N9 plasmid, 1  μL left primers, and 1  μL right primers. The chip containing the sample was then placed on the device. We set up the thermo-cycling in the script to initiate a PCR reaction that was made of 40 cycles of 95 °C  for 15 s and 60 °C for 35 s.

Since the fluorescent dye binds to all double-stranded DNA, it is necessary to analyze the melting curve after each reaction to confirm the specificity of the PCR assay. When the two-strand DNA unfolds, the fluorochrome is freed and the fluorescence signal decreases. According to this principle, the curves for the PCR products without dimer only have one peak, whereas the melt curves of the products containing dimer have two peaks. Our PCR system can also record the melting curve of the product after the reaction. In the script, the melting temperature step can be set. In our experiment, we used a 0.5-degree step, a 3-s lighting time, and 15-s stage for each temperature.

## 3. Results and Discussion

### 3.1. Bubble Elimination

DNA denatures at 95 °C. Such a high temperature causes the formation of bubbles and leads to the failure of the PCR reaction. In many approaches, droplets are placed on the surface of the substrate and sealed with mineral oil [27,28,29]. However, the success of this approach depends on the hydrophobicity of the chip surface. The surface treatment of the microfluidic chip requires the use of PVD which increases the cost of the experiment. Although PVD can be replaced by a relatively less expensive vacuum dryer, the treatment time will be long (generally more than 5 days). Additionally, the hydrophobicity decreases over time, so the chip must be used as soon as possible.

We compared two different chip models and proposed a scheme for bubble suppression. First, we used a vacuum oven to create a hydrophobic wafer. Figure 2(c2) showed that 10 μL droplets of mineral oil were dropped onto the silicon and 2 μL of sample was dripped into the mineral oil. We encountered several issues during our experiments. First, it was difficult to inject the droplet into the center of the mineral oil. If the droplet is off-center (Figure 2(c3)), the sample will evaporate when heated. Second, the sample will evaporate if the silicon wafer is not hydrophobic enough (Figure 2(c4)) and bubbles are generated (Figure 2(c5)). This greatly limits the stability of the instrument.

To solve these problems, we adopted a new microchip structure and optimized the injection sequence. This new structure avoided many problems caused by the injection deviation and the insufficient hydrophobicity, like sample volatilization, thus optimizing the order of injection and also avoiding the formation of bubbles. After the chip was made, sample were added to the chip. In this step, 2.5 μL of mineral oil (Sigma M8410, Sigma-Aldrich, Mo, USA) was injected into the hole and 1 μL was sucked out with a pipette. The purpose of this step is to moisten the inner wall of the hole to prevent the sample from sticking to it. If the sample contacts the PDMS, bubbles will generate during the temperature cycle. Some areas on the inner wall of the pipeline that are prone to bubbles are filled by adding oil, thereby suppressing the generation of bubbles. Then, 1.5 μL sample were infused into the hole by pipette. Finally, the hole was sealed with 1.5 μL of mineral oil to prevent the reagents from volatilzing. Figure 2(c1) shows the droplet after the reaction on the homemade chip. The amount of PDMS used is not set and can be adjusted depending on the requirements. If the PDMS is too thick, it is difficult to inject the samples, and it is easy to produce bubbles during the injection. If the PDMS is too thin, less sample can be added. The size of the PDMS cut can also be changed according to the size of the glass sheet. All the experimental operations are carried out in a normal environment, not in a controlled environment (clean room).

### 3.2. Temperature Cycling

The thermo-cycling program in our experiments was 40 cycles at 95 °C for 15 s, and at 60 °C for 35 s. The temperature variation is an important aspect of the PCR devices. To evaluate the temperature volution, we recorded and drew the temperature change of the PCR instrument. The average heating and cooling rates of the temperature control system were 1.2 °C/s and 1.3 °C/s, respectively. The experimental results showed that the reaction time of the PCR device was 83 min, which is similar to that of a commercial qPCR instrument (Bio-Rad CFX Connect, Bio-Rad, California, USA).

**Figure 3:**
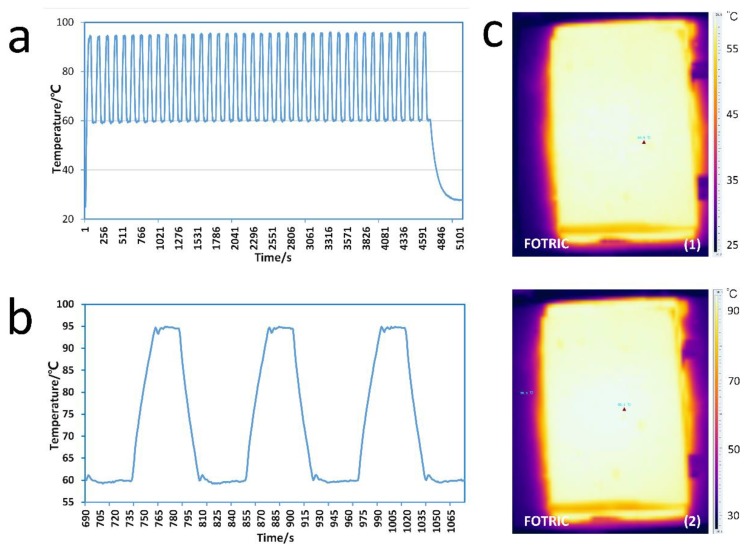
(**a**): Temperature change during PCR. (**b**): Temperature change over two cycles. (**c**): Infrared image of the upper surface of the glass at (1) 60 °C and at (2) 95 °C.

The PCR instruments can feed back the temperature of the chip because of the temperature sensor embedded in the aluminum plate. The temperature sensor measured the temperature at a certain point. To assess the accuracy of the temperature measured, we used an infrared thermal imaging camera to check the temperature of the glass when the instrument was running. Figure 3(c2) shows that the temperature of the glass ranged from 94 °C to 95 °C with a coefficient of variation (CV) of 2.106% when the temperature was set to 95 °C, and from 59 °C to 60 °C with a CV of 2.669% when the temperature was set to 60 °C, which displays a good thermal uniformity.

### 3.3. Fluorescence Imaging

The detection in qPCR relies on the fluorescence of the samples. In this experiment, an LED was used as the excitation light, and cameras (Canon series) were used to collect the fluorescence. To keep the camera in focus, we placed it horizontally above the chip and the excitation light was tilted by 45° to illuminate the chip. Because of the wide spectral characteristics of the LED, we used a narrow-band filter (470–30 nm; Beijing, China). An emission filter (540–20 nm; Beijing, China) was installed in front of the mirror cylinder to avoid the influence of stray light on the fluorescence collected by the camera. In order to reduce the bleaching of the sample fluorescence, we coupled the excitation lighting with the camera shooting and the temperature change through the Python script. Based on the photograph time statistics, the script achieves an excellent coupling of the temperature and the photograph.

**Figure 4:**
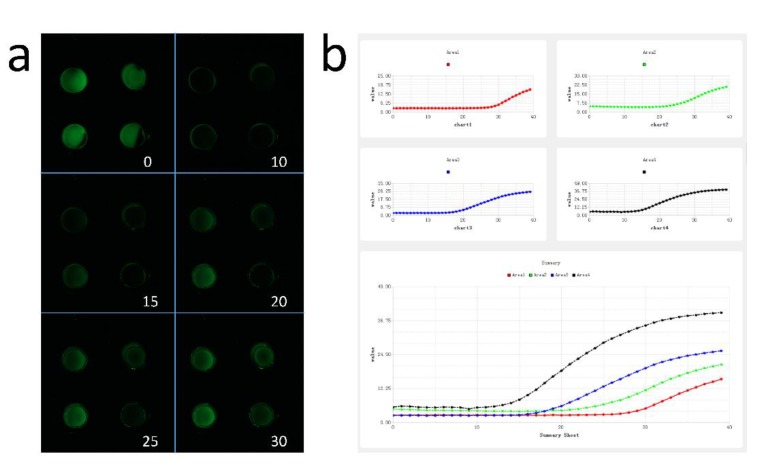
Fluorescence imaging of PCR and results of the software analysis. (**a**): fluorescence images in the 0th (before heating), 10th, 15th, 20th, 25th and 30th cycles. The fluorescence intensity of the stain at room temperature is very strong. The fluorescence intensity of the sample at room temperature before the reaction does not show the concentration of the target gene. (**b**): script processing of the fluorescence imaging.

In polymerase chain reaction, the amount of product obtained in the extension stage is very important for the subsequent analysis. Therefore, the time at which the picture is taken must be controlled. If the photograph is taken too early, the extension is not finished and the brightness of the fluorescence will be lower. If the photograph is taken too late, some of the DNA double-strands will be dissociated when the reaction temperature increases. This leads to a lower fluorescence signal. Therefore, we took the photograph in the last two seconds of the low temperature cycle. In the script analyzing the experimental results, we only need to number the pictures taken by the camera in order, import the folder where the pictures are located into the script, and select the range of droplets. The average brightness data of the selected area are automatically obtained and plotted. This can be exported for further analysis of Ct values. The GUI is shown in Figure 4.

### 3.4. PCR Amplification

**Figure 5:**
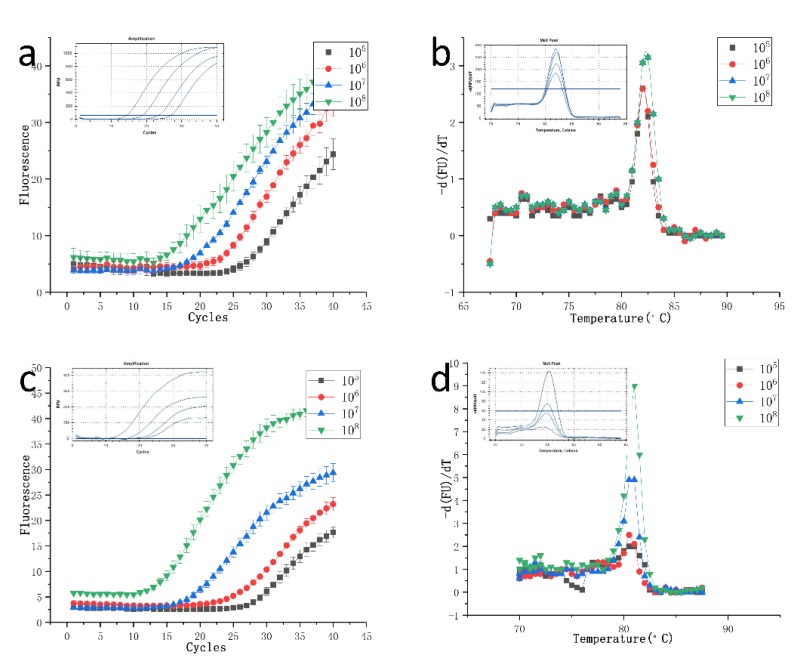
Comparison of the amplification and dissolution curves of H7N9 and pGEM-3Zf (+)1 with those obtained on a commercial instrument. Curves obtained by the real-time PCR amplification system under the same conditions are shown in the upper left of each figure. (**a**): The amplification curves of H7N9 from the homemade system are shown. (**b**): The melt curve analysis of the H7N9 obtained from the homemade system. The first derivative change in fluorescence intensity as a function of temperature is shown. (**c**): The amplification curves of H7N9 from the homemade system are shown. (**d**): The melt curve analysis of the H7N9 obtained from the homemade system. The first derivative change in fluorescence intensity as a function of temperature is shown.

H7N9 characterizes a new type of bird flu and was first detected in Shanghai and Anhui in late March 2013. The incubation period for human infection is generally less than 7 days. Patients generally present flu-like symptoms, and severe patients rapidly show signs of severe pneumonia and dyspnea [33]. Acute respiratory distress syndrome, shock, disturbance of consciousness, and acute kidney injury will develop rapidly [34]. We used our device to detect DNA fragments of H7N9. The Ct values given by qPCR (Bio-Rad CFX Connect) were 14.34, 18.84, 23.44, and 26.94, and the Ct values calculated by the homemade system were 12.03, 16.64, 23.56, and 27.68. In order to avoid the contingency of a single experiment, two repetitive experiments were performed, and the standard deviation was calculated by using the data of three experiments, and the results were showed in Figure 5a. Figure 5 shows that our results were consistent with that of a commercial PCR instrument (Bio-Rad CFX Connect, Bio-Rad, CA, USA). We acquired melting curves to determine that the reaction product was the amplified product. The results obtained by our instrument (Figure 5b) are the same as those obtained by the commercial PCR instrument. Only a single peak corresponding to the PCR product was observed. The amplicon is clean and specific. This confirms the accuracy of our system. We used the device to amplify a pGEM-3Zf (+)1 plasmid to further validate our device. Through repeated experiments, many times both the amplification curve and the Ct value were basically consistent with the commercial instrument. On the other hand, the melt curve is the same as the result of the real-time PCR amplification system, both of which are around 80 °C, showing the peak of the melting point curve.

## 4. Conclusions

In this paper, we have designed and built a qPCR instrument consisting of a TEC and a camera. It achieved the high stability amplification of H7N9 and PGEM through the combination of a glass surface and a PDMS carrier. We increased the automation level of the instrument by writing a script in Python. The device can achieve a high success rate for PCR. Most importantly, the technology and the elements of the device are available through open access, which greatly reduces the technical barriers. Our results show that the qPCR instrument built can achieve the same results as commercial PCR instruments, but at a much lower cost. Our inexpensive and tractable PCR instrument has great potential for practical applications. There are still some disadvantages of the chip, including the potential for improper operation to produce bubbles in the reaction process, which would cause the sample to pollute the environment. In the future, we will actively improve the structure to make microchips more applicable.
